# Expression of vascular endothelial growth factor (VEGF) and its mRNA in uterine cervical cancers

**DOI:** 10.1038/sj.bjc.6690428

**Published:** 1999-05

**Authors:** J Fujimoto, H Sakaguchi, R Hirose, S Ichigo, T Tamaya

**Affiliations:** Department of Obstetrics and Gynecology, Gifu University School of Medicine, 40 Tsukasa-machi, Gifu City, 500-8705, Japan

**Keywords:** VEGF, neovascularization, cervical cancer

## Abstract

To know the potential of growth, invasion and metastasis of uterine cervical cancer associated with neovascularization, localization of vascular endothelial growth factor (VEGF) and microvessel density in tumours were determined by immunohistochemical staining, the levels of VEGF subtypes were determined by Western blot analysis and by a sandwich enzyme immunoassay, and the levels of VEGF subtype mRNAs were determined by reverse transcription polymerase chain reaction (RT-PCR) – Southern blot analysis in uterine cervical cancers. The relation between VEGF subtype expressions and microvessel density, histological types and clinical stages of uterine cervical cancers was analysed. The expression of VEGF was seen dominantly in the cancer cells, and correlated with microvessel density in uterine cervical cancers. Among the four subtypes of VEGF, the populations of VEGF_165_ and VEGF_121_ were dominant in normal uterine cervices and uterine cervical cancers. The levels of VEGF and VEGF_165_ and VEGF_121_ mRNAs were remarkably higher in some stage II and III/IV adenocarcinomas of the cervix than in other cases, including normal cervices. Therefore, the elevation of VEGF_165_ and VEGF_121_ might contribute to the relatively late advancing via angiogenic activity in some adenocarcinomas of the cervix. © 1999 Cancer Research Campaign


*British Journal of Cancer* (1999) **80**(5/6), 827–833
© 1999 Cancer Research Campaign
Article no. bjoc.1998.0428

### Expression of vascular endothelial growth factor (VEGF) and its mRNA in uterine cervical cancers

**J Fujimoto, H Sakaguchi, R Hirose, S Ichigo and T Tamaya**

Department of Obstetrics and Gynecology, Gifu University School of Medicine, 40 Tsukasa-machi, Gifu City 500-8705, Japan

**Summary** To know the potential of growth, invasion and metastasis of uterine cervical cancer associated with neovascularization, localization of vascular endothelial growth factor (VEGF) and microvessel density in tumours were determined by immunohistochemical staining, the levels of VEGF subtypes were determined by Western blot analysis and by a sandwich enzyme immunoassay, and the levels of VEGF subtype mRNAs were determined by reverse transcription polymerase chain reaction (RT-PCR) – Southern blot analysis in uterine cervical cancers. The relation between VEGF subtype expressions and microvessel density, histological types and clinical stages of uterine cervical cancers was analysed. The expression of VEGF was seen dominantly in the cancer cells, and correlated with microvessel density in uterine cervical cancers. Among the four subtypes of VEGF, the populations of $VEGF_{165}$ and $VEGF_{121}$ were dominant in normal uterine cervices and uterine cervical cancers. The levels of VEGF and $VEGF_{165}$ and $VEGF_{121}$ mRNAs were remarkably higher in some stage II and III/IV adenocarcinomas of the cervix than in other cases, including normal cervices. Therefore, the elevation of $VEGF_{165}$ and $VEGF_{121}$ might contribute to the relatively late advancing via angiogenic activity in some adenocarcinomas of the cervix.

**Keywords:** VEGF; neovascularization; cervical cancer

Neovascularization is essential for growth of, and nutrition to, solid tumours greater than 2 mm in diameter (Folkman, 1985). Unorganized basement membrane of new capillary endothelial cells allows intravasation of tumour cells, and a high density of microvessels in tumours is associated with their expansion and invasiveness (Srivastava et al, 1988; Weidner et al, 1991; Macchiarini et al, 1992; Wakui et al, 1992; Weidner et al, 1993). Neovascularization consists of the following steps: dissolution of basement membrane by proteases which are released from the tumour or host cells activated by tumour-derived angiogenic factors, migration and proliferation of endothelial cells, and capillary-tube formation (Folkman and Haudenschild, 1980). Angiogenic factors stimulate the corresponding steps of neovascularization.

Generally speaking, angiogenic factors have been identified as follows: basic fibroblast growth factor (bFGF), VEGF, placenta growth factor (PlGF), epidermal growth factor (EGF), transforming growth factor (TGF)-α, TGF-β, platelet-derived growth factor (PDGF), platelet-derived endothelial cell growth factor (PD-ECGF), hepatocyte growth factor (HGF), tumour necrosis factor (TNF)-α, pleiotropin, proliferin, angiogenin, oestradiol, interleukin (IL)-8, etc. Among them, main factors induced from tumour cells are bFGF, VEGF, PD-ECGF and IL-8. VEGF was initially recognized as a vascular permeability factor (34–42 kDa) which induced tumour ascites (Senger et al, 1983). Afterwards, VEGF was identified as a vascular permeability factor that is active in increasing blood vessel permeability, endothelial cell growth and angiogenesis (Leung et al, 1989). On the other hand, it was described as having a direct-acting mitogen specific for vascular endothelial cells (Keck et al, 1989). VEGF is expressed in tissues with rapid vascular endothelial turnover, i.e. reproductive organs such as ovary, uterus and placenta (Garrido et al, 1993; Jackson et al, 1994; Li et al, 1994), and ovarian and uterine cervical and endometrial cancers (Olson et al, 1994; Guidi et al, 1995; Guidi et al, 1996). In detail, VEGF contributes to angiogenic potential in premalignant changes (Dobbs et al, 1997), invasive carcinoma (Guidi et al, 1995) and adenocarcinoma of the cervix (Tokumo et al, 1998). However, it is unknown which VEGF subtypes (Houck et al, 1991) contribute to angiogenic potential in uterine cervical cancers.

To know the potential of growth, invasion and metastasis of uterine cervical cancer associated with neovascularization, localization of VEGF and microvessel density in tumours were determined by immunohistochemical staining, the levels of VEGF subtypes were determined by Western blot analysis and by a sandwich enzyme immunoassay, and the levels of VEGF subtype mRNAs were determined by reverse transcription polymerase chain reaction (RT-PCR)–Southern blot analysis in uterine cervical cancers and uterine normal cervices as controls. The relation between VEGF subtype expressions and microvessel density, histological types and clinical stages of uterine cervical cancers was analysed.

### PATIENTS AND METHODS

### Patients

Agreements for the following studies were obtained from all patients and the Research Committee for Human Subjects, Gifu University School of Medicine. A total of 150 patients ranging from 32 to 83 years of age underwent hysterectomy for leiomyoma or uterine cervical cancer at the Department of Obstetrics and Gynecology, Gifu University School of Medicine, between March

*Received 10 February 1998*
*Revised 26 October 1998*
*Accepted 4 November 1998*

*Correspondence to:* J Fujimoto

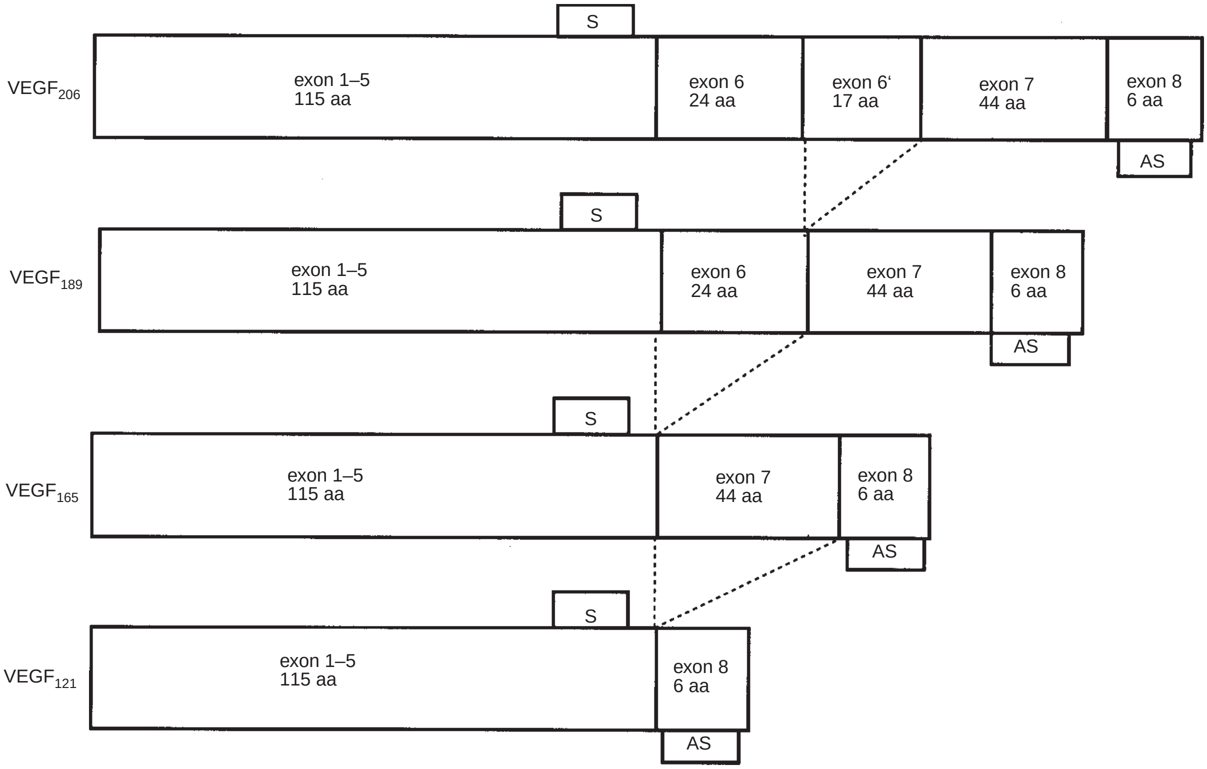

**Figure 1** Schematic representation of human VEGF isoforms. exon 1–5, exons 1, 2, 3, 4 and 5; S, sense primer for human VEGF cDNA; AS, antisense primer for human VEGF

1994 and December 1997. None of the patients received any preoperative therapy. A part of the tissues of uterine cervical cancers and normal uterine cervices as controls was obtained immediately after hysterectomy and snap-frozen in liquid nitrogen to determine the levels of VEGF and its mRNA expression, and a neighbouring part of the tissues was submitted for histopathological study. The clinical staging was determined by International Federation of Obstetrics and Gynecology (FIGO) classification (FIGO News, 1989).

### Immunohistochemistry

Four-micrometer sections were cut from formalin-fixed paraffin-embedded tissue with a microtome and dried overnight at 37°C on a silanized slide (Dako, Carpinteria, CA, USA). Samples were deparaffinized in xylene at room temperature for 80 min and washed with a graded ethanol–water mixture and then with distilled water. The samples for VEGF were soaked in a citrate buffer and then microwaved at 100°C for 10 min, and those for factor VIII-related antigen were treated with 0.3 μg $ml^{-1}$ trypsin in a phosphate buffer at room temperature for 20 min. The protocol for DAKO LSAB2 Kit, Peroxidase (Dako) was followed for each sample. In the described procedure, rabbit anti-human VEGF antigen VEGF(147) (200 μg $ml^{-1}$, Santa Cruz, CA, USA), and rabbit anti-factor VIII-related antigen (Zymed, San Francisco, CA, USA) as the first antibodies were used at dilutions of 1:100 and 1:2 respectively. Vascular density was evaluated with microvessel counting (Maeda et al, 1996). The addition of the first antibody was omitted in the protocol for negative controls.

### Western blot analysis for human VEGF

Tissues (wet weight: 10–20 mg) were homogenized in WB-HB buffer (10 mM) Tris–HCl, pH 7.4, 150 mM sodium chloride (NaCl), 0.5% Triton X-100 and 0.2 mM phenylmethylsulphonyl fluoride) with a Polytron homogenizer (Kinematics, Lucerne, Switzerland). The protein concentration of samples was measured by the method of Bradford (Bradford, 1976). Each sample (25 μl) containing 100 μg of protein was added to 25 μl of a sample buffer (12.5 mM Tris–HCl, pH 6.8, 2% glycerol, 0.4% sodium dodecyl sulphate (SDS) and 1.25% 2-mercaptoethanol) and analysed by 7.5% SDS polyacrylamide gel electrophoresis (SDS-PAGE) under non-reducing conditions. The gel was transferred to a nitrocellulose membrane (Hybond ECL Western; Amersham, Arlington Heights, IL, USA). The membrane was blocked with 5% milk (from dehydrate) in a blocking buffer (20 mM Tris–HCl, pH 7.6, 137 mM NaCl and 0.1% Tween-20), incubated with rabbit anti-human VEGF antibody (1:1000) (Oncogene Research Products, Cambridge, MA, USA), washed, and then incubated with peroxidase-linked species-specific whole antibody, anti-rabbit immunoglobulin from donkey (1:2000) (Amersham, Buckinghamshire, UK). Specific bands were detected with electrochemiluminescence (ECL) reagent (Amersham, Arlington Heights, IL, USA), and X-ray film was exposed on the membrane at room temperature for 10 min.

 *© Cancer Research Campaign 1999*

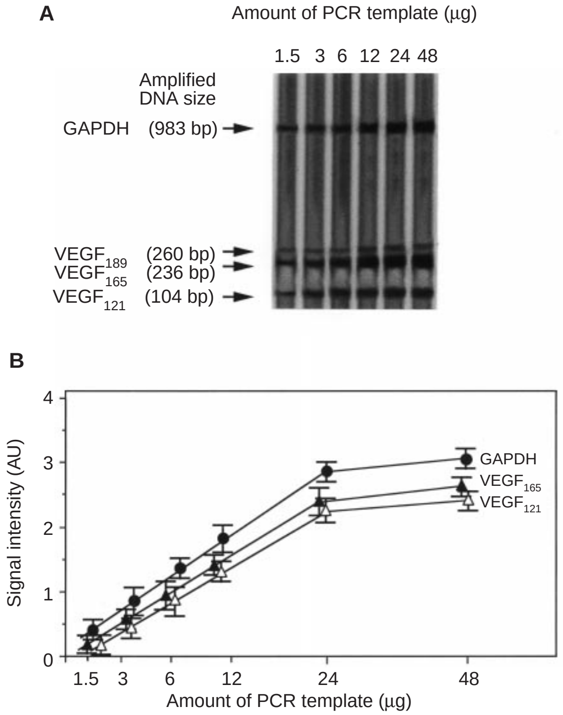

**Figure 2** Signal intensity curve for VEGF mRNA level in a series of reverse transcribed-total RNA of normal uterine cervix by RT-PCR–Southern blot analysis. A series amount of PCR templates were prepared from reverse transcribed total RNA in uterine normal cervices. PCR–Southern blots were carried out as described in Materials and Methods. (**A**) A representative Southern blot for the expressions of VEGF and GAPDH mRNAs. (**B**) The levels of VEGF mRNA expression in normal uterine cervix were assigned as arbitrary units/GAPDH mRNA (AU/GAPDH mRNA). Data are the mean$\pm$ s.d. of nine determinations

### Enzyme immunoassay for determination of human VEGF antigen

All steps were carried out at 4°C. Tissues (wet weight: 10–20 mg) were homogenized in HG buffer (5 mM Tris–HCl, pH 7.4, 5 mM NaCl, 1 mM calcium chloride, 2 mM ethyleneglycol-bis-[β-aminoethyl ether]-N,N,N′,N′-tetraacetic acid, 1 mM magnesium chloride ($MgCl_2$), 2 mM dithiothreitol (DTT), 25 μg $ml^{-1}$ aprotinin and 25 μg $ml^{-1}$ leupeptin) with a Polytron homogenizer (Kinematics). This suspension was centrifuged in a microfuge at 12 000 rpm for 3 min to remove the nuclear pellet. The protein concentration of samples was measured by the method of Bradford to standardize VEGF antigen levels (Bradford, 1976).

VEGF antigen levels in the samples were determined by a sandwich enzyme immunoassay using a Human VEGF Assay Kit-IBL (Immuno Biological Laboratories, Gunma, Japan). The levels of VEGF were standardized with the corresponding cellular protein concentrations.

### RT-PCR to amplify VEGF mRNA

The oligodeoxynucleotides of specific primers in PCR were synthesized according to the published information (cDNA for VEGF (Houck et al, 1991) as shown in Figure 1 and glyceraldehyde-3-phosphate dehydrogenase (GAPDH) (Arcari et al, 1984)) as follows:

sense primer for VEGF mRNA: 5′-AGGCCAGCACATAG-GAGAGA-3′ (in exon 4)
antisense primer for VEGF mRNA: 5′-ACCGCCTCG-GCTTGTCACAT-3′ (in exon 8)
sense primer for GAPDH mRNA: 5′-TGAAGGTCGGAGT-CAACGGATTTGGT-3′ (in exon 2)
antisense primer for GAPDH mRNA: 5′-CATGTGGGCCAT-GAGGTCCACCAC-3′ (in exon 8).

Total RNA was isolated from the cells by the acid guanidium thiocyanate–phenol–chloroform extraction method (Chomczynski and Sacchi, 1987). Total RNA was reverse transcribed with Moloney murine leukaemia virus reverse transcriptase (MMLV-RTase, 200 units, Gibco BRL, Gaithersburg, MD, USA) in a buffer of 20 mM Tris–HCl, pH 8.4, 50 mM potassium chloride (KCl), 2.5 mM $MgCl_2$, 0.1 mg $ml^{-1}$ bovine serum albumin, 10 mM DTT and 0.5 mm deoxynucleotides to generate cDNAs using random hexamer (50 ng, Gibco BRL) at 37°C for 60 min. The RT reaction mixture was heated at 94°C for 5 min to inactivate MMLV-RTase.

A B

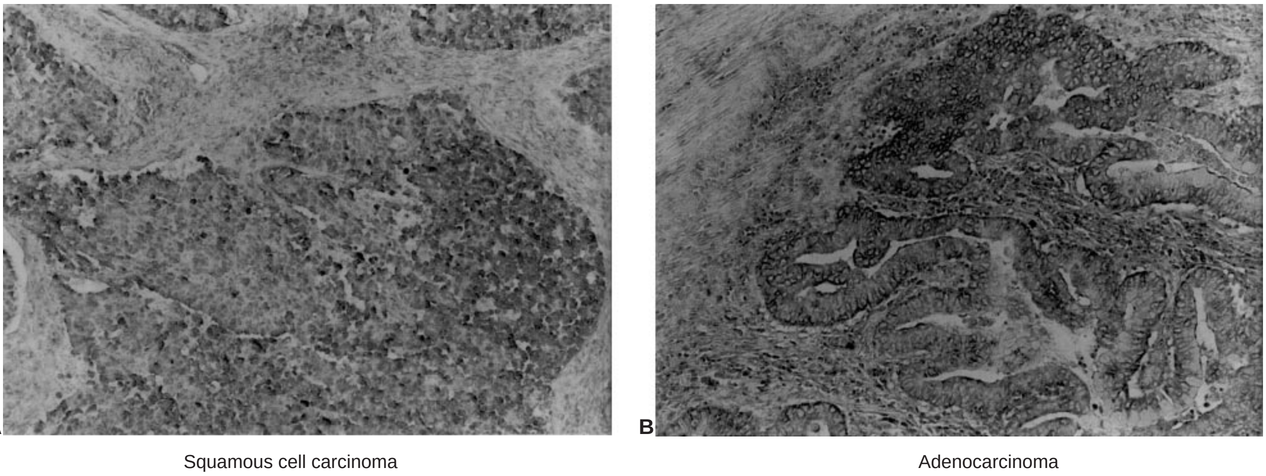

Squamous cell carcinoma Adenocarcinoma

**Figure 3** Immunohistochemical staining for VEGF in uterine cervical cancers. Positive staining is seen dominantly in the cytoplasm of the cancer cells, and faintly in interstitial cells. The intensity of VEGF staining was significantly stronger in adenocarcinomas (**B**) than in squamous cell carcinomas (**A**) (original magnification ×200)

*© Cancer Research Campaign 1999* 

Ten cycles of PCR for VEGF mRNA, consisting of denaturation for 1 min at 94°C, annealing for 1 min at 55°C, and extension for 1 min at 72°C, were carried out with reverse transcribed cDNA, 0.1 μM specific primers and Vent DNA polymerase (New England Biolabs, Beverly, MA, USA) in a buffer of 10 mM KCl, 20 mM Tris–HCl, pH 8.8, 10 mM $(NH_4)_2SO_4$, 2 mM $MgSO_4$, 0.1% Triton X-100 and 0.15 mM deoxynucleotide phosphates using the IWAKI thermal sequencer TSR-300 (Iwaki Glass, Tokyo, Japan). Additionally, 23 cycles of PCR for VEGF and GAPDH mRNAs as an internal standard were done in the same manner.

The signal intensity curve for mRNA expression is necessary for an accurate measurement of the mRNA by RT-PCR. A series of PCR templates (1.5 μg, 3 μg, 6 μg, 12 μg, 24 μg and 48 μg reverse transcribed-total RNA) were prepared to get the signal intensity curve of the mRNA levels on RT-PCR–Southern blot. The signal intensity curve for VEGF and GAPDH mRNA levels ranging from 1.5 μg to 24 μg reverse transcribed-total RNA of normal uterine cervix by RT-PCR–Southern blot was linear (Figure 2). Therefore, semiquantative alternation of VEGF mRNA levels standardized with GAPDH mRNA levels was thought to be reliable. Additionally, the template for negative controls of RT-PCR was not reverse transcribed. The Southern blot revealed no band in the negative control for each sample (data not shown).

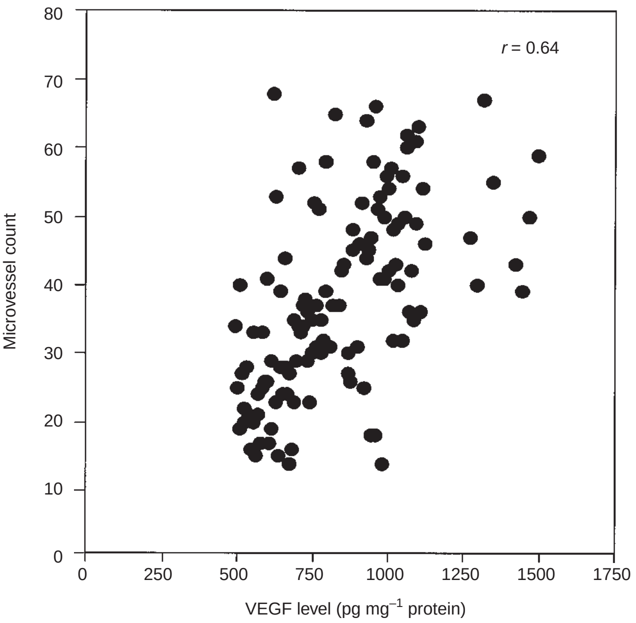

**Figure 4** Correlation between VEGF level and microvessel count

### Southern blot analysis for quantities of VEGF mRNA expression

PCR products were applied to 1.2% agarose gel, and electrophoresis was performed at 50–100 V. PCR products were capillary-transferred to an Immobilon transfer membrane (Millipore Corp., Bedford, MA, USA) for 16 h. The membrane was dried at 80°C for 30 min, and was UV-irradiated to tightly fix PCR products. PCR products on the membrane were prehybridized in a buffer of 1 M NaCl, 50 mM Tris–HCl pH 7.6 and 1% SDS at 42°C for 1 h, and hybridized in the same solution with the biotinylated oligodeoxynucleotide probes synthesized from the sequences of VEGF and GAPDH cDNAs between the specific primers at 65°C overnight as follows: probe for VEGF gene: 5′-CAGCACAA-CAAATGTGAATG-3′ (in exon 4); probe for GAPDH gene: 5′-GCTCACTGGCATGGCCTTCC-3′ (in exon 8).

Specific bands hybridized with the biotinylated probes were detected with Plex Luminescent Kits (Millipore Corp.), and X-ray film was exposed on the membrane at room temperature for 10 min. The quantification of Southern blot was carried out with Bio Image (Millipore, Ann Arbor, MI, USA). The intensity of specific bands was standardized with that of GAPDH mRNA.

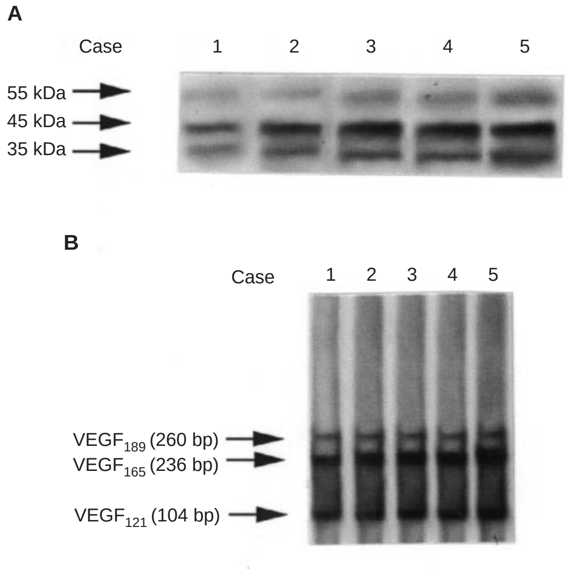

**Figure 5** Western blot analysis for the expression of VEGF and Southern blot analysis for the expression of VEGF mRNA in uterine cervical cancers. Case 1: normal uterine cervix; Case 2: Stage I, LNK; Case 3: Stage II, K; Case 4: Stage III, SNK; Case 5: Stage IV, AD. K, keratinizing squamous cell carcinoma; LNK, large cell non-keratinizing squamous cell carcinoma; SNK, small cell non-keratinizing squamous cell carcinoma; AD, adenocarcinoma. Histological types and clinical stages of uterine cervical cancers are according to International Federation of Gynecology and Obstetrics [FIGO] (FIGO News, 1989)

### Statistics

The levels of VEGF and its mRNA were measured from three parts of the same tissue in triplicate. Statistical analysis was performed with Student's $t$-test; differences were considered significant when $P$ was less than 0.05.

### RESULTS

Immunohistochemical staining for VEGF ($n$ = 120) was carried out to study VEGF localization in the tumours, and the strength of staining was correlated with VEGF levels measured by a sandwich enzyme immunoassay. As shown in Figure 3, positive staining is seen dominantly in the cytoplasm of the cancer cells, and faintly in interstitial cells. The intensity of VEGF staining was significantly stronger in adenocarcinomas than in squamous cell carcinomas. There was a significant correlation between VEGF levels and microvessel count, as shown in Figure 4 ($y = 0.034\ x + 9.049$, $r = 0.64$.)

To analyse which subtypes of VEGF (as shown in Figure 5) induce angiogenesis, the following studies were carried out. In

 © Cancer Research Campaign 1999

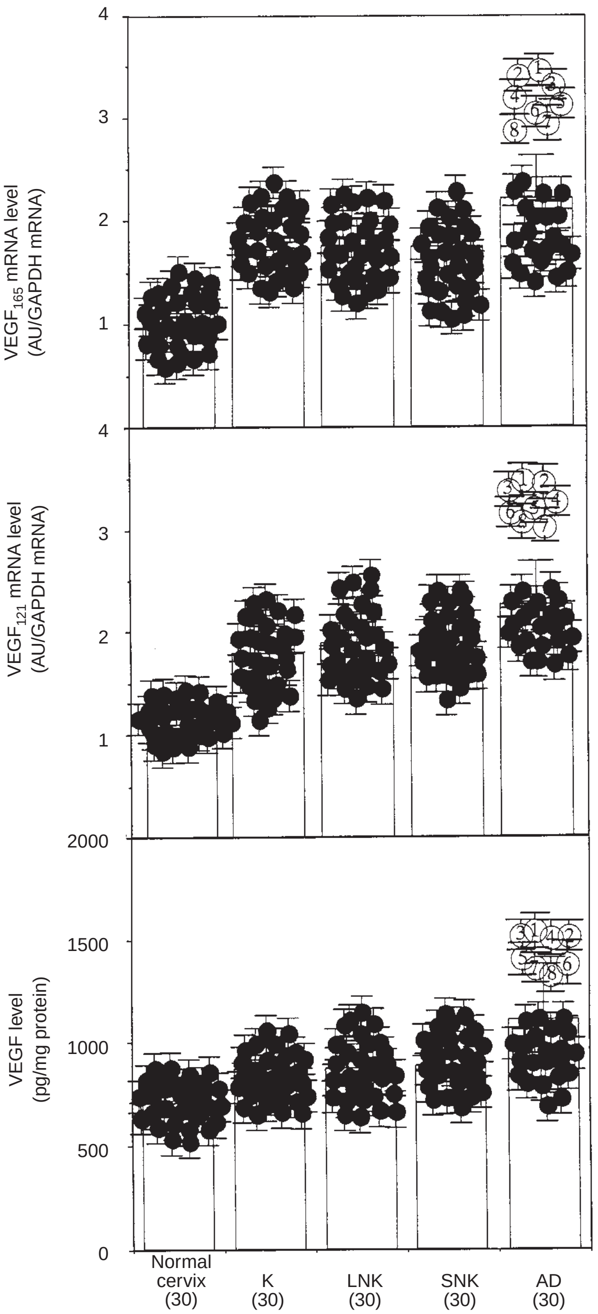

**Figure 6** Levels of VEGF and its mRNA in uterine cervical cancers classified according to histological types. The levels of VEGF and its mRNA were determined by a sandwich enzyme immunoassay and RT-PCR–Southern blot analysis respectively. The mRNA level in normal uterine cervices as controls was assigned as AU/GAPDH mRNA. Histological types of uterine cervical cancers are according to FIGO. Each result is the mean ± s.d. of nine determinations. The number in ○ indicates each case in which VEGF and its mRNA were expressed relatively high

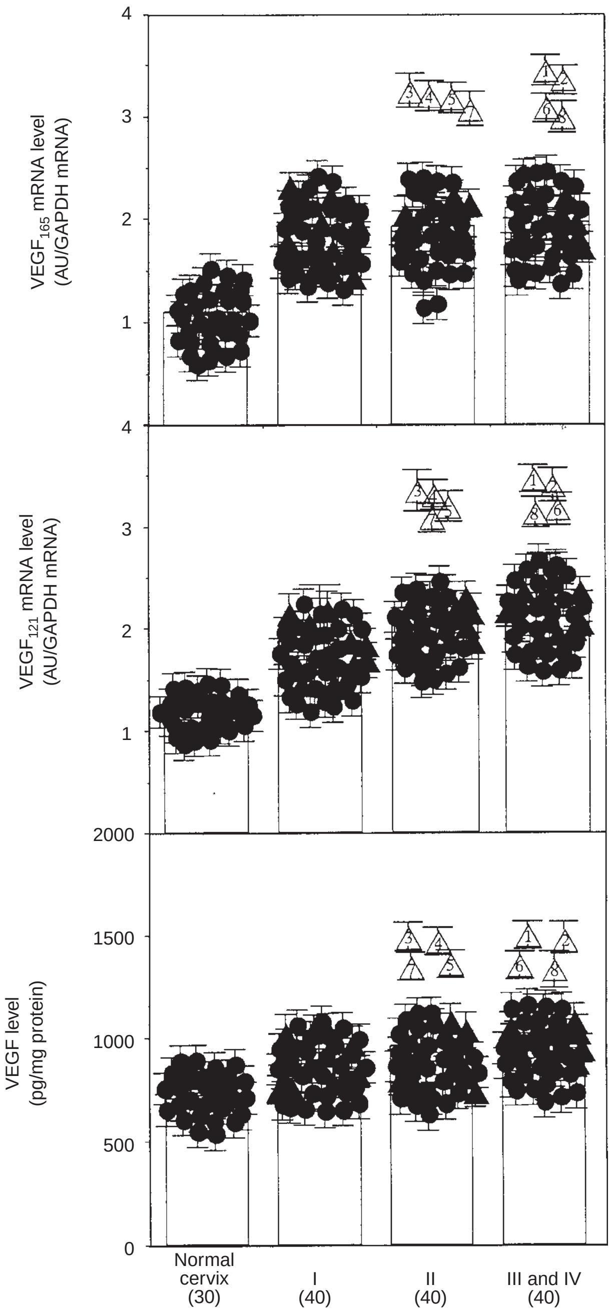

**Figure 7** Levels of VEGF and its mRNA in uterine cervical cancers classified according to clinical stages. Clinical stages of endometrial cancer are according to FIGO. Each result is the mean ± s.d. of nine determinations. △ and ▲, adenocarcinoma of the cervix. The number in △ indicates each case in which VEGF and its mRNA were expressed relatively high uterine cervical cancers and normal uterine cervices, mainly VEGF$_{165}$ and VEGF$_{121}$ and their mRNAs were detected by Western blot and RT-PCR–Southern blot respectively. VEGF$_{189}$ and its mRNA were only remarkably faintly detected, and VEGF$_{206}$ and its mRNA were not detected. The two main subtypes and their mRNAs expressed in uterine cervical cancers and normal uterine cervices are shown in parallel in Figure 5. The simultaneous determinations of total VEGF level by a sandwich enzyme immunoassay and subtype mRNAs with RT-PCR–Southern blot is believed to provide enough information to reliably identify the population of subtype protein expressions.

Among the four subtypes of VEGF, the populations of VEGF$_{165}$ and VEGF$_{121}$ were dominant in normal uterine cervices and uterine cervical cancers. There was no significant difference in the levels of VEGF, and VEGF$_{165}$ and VEGF$_{121}$ mRNAs among normal uterine cervices and uterine cervical cancers classified according

*© Cancer Research Campaign 1999*

to histological types. However, they were remarkably higher in some adenocarcinomas of the cervix than in other cases, including normal cervices (Figure 6). Similarly, there was no significant difference in their levels among normal uterine cervices and uterine cervical cancers classified according to clinical stages. However, their levels were remarkably higher in some stages II and III/IV adenocarcinomas of the cervix than in other cases, including normal cervices (Figure 7).

### DISCUSSION

Newly developed capillary network formation from the original vessel is designated as neovascularization. Generally, turnover of capillary endothelial cells is extremely slow, to the order of months or years in physiological neovascularization, while the turnover in ovary and uterine endometrium is rapidly altered along with ovarian cycle. The turnover with malignant transformation becomes rapid, which might contribute to the acceleration of tumour growth (Denekamp, 1984).

Expression of the tumour cell-derived angiogenic factors, bFGF, VEGF, PD-ECGF and IL-8, may be specific for each tumour and dependent on the process of tumour growth and spreading. The levels of bFGF and its mRNA were higher in advanced primary uterine cervical cancers, regardless of histological type (Fujimoto et al, 1997). The levels of PD-ECGF and its mRNA were higher and in a wider range in uterine cervical cancers, especially squamous cell carcinomas, regardless of clinical stage (Fujimoto et al, 1999). VEGF, secreted from many tumours, does not contribute to tumour growth via an autocrine pathway to tumour cells, but via a paracrine pathway to surrounding microvessels (Berkman et al, 1993). Furthermore, it facilitates metastasis via neovascularization (Warren et al, 1995). In the present study, it is demonstrated that VEGF dominantly expresses in the cancer cells, and VEGF levels correlate to microvessel density in uterine cervical cancers.

Among VEGF subtypes, $VEGF_{189}$ is closely associated with progression of non-small-cell lung cancer (Oshika et al, 1998). Coexpression of $VEGF_{121}$, $VEGF_{165}$ and especially $VEGF_{189}$ was correlated with liver metastasis and poor prognosis in colon cancer (Tokunaga et al, 1998). Only $VEGF_{165}$ protein was detected in normal and malignant breast tissues (Scott et al, 1998). $VEGF_{165}$ was elevated in all stages of ovarian carcinoma via angiogenic activity (Fujimoto et al, 1998*a*). Intracerebral tumour-associated haemorrhage was caused by overexpression of $VEGF_{121}$ and $VEGF_{165}$ but not $VEGF_{189}$ (Cheng et al, 1997). $VEGF_{165}$ and $VEGF_{121}$ in some uterine endometrial cancers contribute to the early process of advancing of malignancy via angiogenic activity (Fujimoto et al, 1998*b*). In the present study, the populations of $VEGF_{165}$ and $VEGF_{121}$ were dominant in uterine cervical cancers. $VEGF_{145}$ expression was demonstrated in several cell lines derived from the female reproductive system, and might contribute unique biological properties distinct from those of previously characterized VEGF isoforms (Poltorak et al, 1997). We noticed that in approximately 10% of cases the band of $VEGF_{165}$ was relatively thick, and might involve the isoform $VEGF_{145}$. However, in uterine cervical cancers, the dominant expressed isoforms were definitely $VEGF_{165}$ and $VEGF_{121}$. Therefore, the manner of VEGF subtype expression might depend upon tissue specificity, and the levels of $VEGF_{165}$ and $VEGF_{121}$ might be essential to total angiogenic activity supplied from VEGFs. The levels of VEGF and $VEGF_{165}$ and $VEGF_{121}$ mRNAs were higher in some relatively advanced adenocarcinomas of the cervix. Therefore, the elevation of $VEGF_{165}$ and $VEGF_{121}$ might contribute to the relatively late advancing via angiogenic activity in some adenocarcinomas of the cervix. This indicates that angiogenic inhibitors (Folkman and Ingber, 1987; Fujimoto et al, 1989; Ingber et al, 1990) on the new capillary formation might be effective as therapy for some adenocarcinomas of the cervix apart from a direct anti-tumoural effect on cancer cells.

### REFERENCES

Arcari P, Martinelli R and Salvatore F (1984) The complete sequences of a full length cDNA for human liver glyceraldehyde-3-phosphate dehydrogenase: evidence for multiple mRNA species. *Nucleic Acids Res* **12**: 9179–9189

Berkman RA, Merrill MJ, Reinhold WC, Monacci WT, Saxena A, Clark WC, Robertson JT, Ali IU and Oldfield EH (1993) Expression of vascular permeability factor/vascular endothelial growth factor gene in central nervous system neoplasms. *J Clin Invest* **91**: 153–159

Bradford M (1976) A rapid and sensitive method for the quantitation of microgram quantities of protein utilizing the principle of protein-dye binding. *Anal Biochem* **72**: 315–323

Cheng SY, Nagane M, Huang HS and Cavenee WK (1997) Intracerebral tumor-associated hemorrhage caused by overexpression of the vascular endothelial growth factor isoforms VEGF121 and VEGF165 but not VEGF189. *Proc Natl Acad Sci USA* **94**: 12081–12087

Chomczynski P and Sacchi N (1987) Single-step method of RNA isolation by acid guanidium thiocyanate–phenol–chloroform extraction. *Anal Biochem* **162**: 156–159

Denekamp J (1984) Vascular as a target for tumor therapy. *Prog ApplMicrocirc* **4**: 28–38

Dobbs SP, Hewett PW, Johnson IR, Carmichael J and Murray JC (1997) Angiogenesis is associated with vascular endothelial growth factor expression in cervical intraepithelial neoplasia. *Br J Cancer* **76**: 1410–1415

FIGO News (1989) Int J Gynecol Obstet **28**: 189–193

Folkman J (1985) Tumor angiogenesis. *Adv Cancer Res* **43**: 175–203

Folkman J and Haudenschild C (1980) Angiogenesis in vitro. *Nature* **288**: 551–556

Folkman J and Ingber DE (1987) Angiostatic steroids. *Ann Surg* **206**: 374–383

Fujimoto J, Hosoda S, Fujita H and Okada H (1989) Inhibition of tumor angiogenesis activity by medroxyprogesterone acetate in gynecologic malignant tumors. *Invasion Metastasis* **9**: 269–277

Fujimoto J, Ichigo S, Hori M, Hirose R, Sakaguchi H and Tamaya T (1997) Expression of basic fibroblast growth factor and its mRNA in advanced uterine cervical cancers. *Cancer Lett* **111**: 21–26

Fujimoto J, Sakaguchi H, Hirose R, Ichigo S and Tamaya T (1998*a*) Biological implication of the expression of vascular endothelial growth factor in ovarian cancers. *Cancer* **83**: 2528–2533

Fujimoto J, Sakaguchi H, Hirose R and Tamaya T (1998*b*) Expression of vascular endothelial growth factor (VEGF) and its mRNA in uterine endometrial cancers. *Cancer Lett* **134**: 15–22

Fujimoto J, Ichigo S, Sakaguchi H, Hirose R and Tamaya T (1999) Expression of platelet-derived endothelial cell growth factor (PD-ECGF) and its mRNA in uterine cervical cancers. *Br J Cancer* **79**: 1249–1254

Garrido C, Saule S and Gospodarowicz D (1993) Transcriptional regulation of vascular endothelial growth factor gene expression in ovarian bovine granulosa cells. *Growth Factor* **8**: 109–117

Guidi AJ, Abu-Jawdeh G, Berse B, Jackman RW, Tognazzi K, Dvorak HF and Brown LF (1995) Vascular permeability factor (vascular endothelial growth factor) expression and angiogenesis in cervical neoplasia. *J Natl Cancer Inst* **87**: 1237–1245

Guidi AJ, Abu-Jawdeh G, Tognazzi K, Dvorak HF and Brown LF (1996) Expression of vascular permeability factor (vascular endothelial growth factor) and its receptors in endometrial cancers. *Cancer* **78**: 454–460

Houck KA, Ferrara N, Winer J, Cachianes G, LI B and Leung DW (1991) The vascular endothelial growth factor family: identification of a fourth molecular species and characterization of alternative splicing of RNA. *Mol Endocrinol* **12**: 1806–1814

Ingber D, Fijita T, Kishimoto S, Sudo K, Kanamaru T, Brem H and Folkman J (1990) Synthetic analog of fumagillin that inhibit angiogenesis and suppress tumour growth. *Nature* **348**: 555–557

Jackson MR, Carney EW, Lye SJ and Knoxritchie JW (1994) Localization of two angiogenic growth factors (PDECGF and VEGF) in human placentae throughout gestation. *Placenta* **15**: 341–353

© Cancer Research Campaign 1999

Keck PJ, Hauser SD, Krivi G, Sanzo K, Warren T, Feder J and Connolly DT (1989) Vascular permeability factor, an endothelial cell mitogen related to PDGF. *Science* **246**: 1309–1312

Leung DW, Cachianes G, Kuang W-J, Goeddel DV and Ferrara N (1989) Vascular endothelial growth factor is a secreted angiogenic mitogen. *Science* **246**: 1306–1309

Li XF, Gregory J and Ahmed A (1994) Immunolocalization of vascular endothelial growth factor in human endometrium. *Growth Factors* **11**: 277–282

Macchiarini P, Fontanini G, Hardin MJ, Squartini F and Angeletti GA (1992) Relation of neovascularization to metastasis of non-small-cell lung cancer. *Lancet* **340**: 145–146

Maeda K, Chung YS, Ogawa Y, Takatsuka S, Kang SM, Ogawa M, Sawada T, Onoda N, Kato Y and Sowa M (1996) Thymidine phosphorylase/platelet-derived endothelial cell growth factor expression associated with hepatic metastasis in gastric carcinoma. *Br J Cancer* **73**: 884–888

Olson TA, Mohanraj D, Carson LF and Ramakrishnan S (1994) Vascular permeability factor gene expression in normal and neoplastic human ovaries. *Cancer Res* **54**: 276–280

Oshika Y, Nakamura M, Tokunaga T, Ozeki Y, Fukushima Y, Hatanaka H, Abe Y, Yamazaki H, Kijima H, Tamaoki N and Ueyama Y (1998) Expression of cell-associated isoform of vascular endothelial growth factor 189 and its prognostic relevance in non-small cell lung cancer. *Int J Oncol* **12**: 541–544

Poltorak Z, Cohen T, Sivan R, Kandelis Y, Spira G, Vlodavsky I, Keshet E and Neufeld G (1997) VEGF145, a secreted vascular endothelial growth factor isoform that binds to extracellular matrix. *J Biol Chem* **272**: 7151–7158

Scott PA, Smith K, Poulsom R, DE Benedetti A, Bicknell R and Harris AL (1998) Differential expression of vascular endothelial growth factor mRNA vs protein isoform expression in human breast cancer and relationship to eIF–4E. *Br J Cancer* **77**: 2120–2128

Senger DR, Galli SJ, Dvorak AM, Perruzzi CA, Harvey VS and Dvorak HS (1983) Tumor cells secrete a vascular permeability factor that promotes accumulation of ascites fluid. *Science* **219**: 983–985

Srivastava A, Laidler P, Davies RP, Horgan K and Hughes L (1988) The prognostic significance of tumor vascularity in intermediate-thickness (0.76–4.0 mm thick) skin melanoma. *Am J Pathol* **133**: 419–423

Tokumo K, Kodama J, Seki N, Nakanishi Y, Miyagi Y, Kaminuma S, Yoshinouchi M, Okuda H and Kudo T (1998) Different angiogenic pathways in human cervical cancers. *Gynecol Oncol* **68**: 38–44

Tokunaga T, Oshika Y, Abe Y, Ozeki Y, Sadahiro S, Kijima H, Tsucida T, Yamazaki H, Ueyama Y, Tamaoki N and Nakamura M (1998) Vascular endothelial growth factor (VEGF) mRNA isoform expression pattern is correlated with liver metastasis and poor prognosis in colon cancer. *Br J Cancer* **77**: 998–1002

Wakui S, Furusato M, Itoh T, Sasaki H, Akiyama A, Kinoshita I, Asano K, Tokuda T, Aizawa S and Ushigome S (1992) Tumor angiogenesis in prostatic carcinoma with and without bone marrow metastasis: a morphometric study. *J Pathol* **168**: 257–262

Warren RS, Yuan H, Matli MR, Gillett NA and Ferrara N (1995) Regulation by vascular endothelial growth factor of human colon cancer tumorigenesis in a mouse of experimental liver metastasis. *J Clin Invest* **95**: 1789–1797

Weidner N, Semple JP, Welch WR and Folkman J (1991) Tumor angiogenesis and metastasis: correlation in invasive breast carcinoma. *N Engl J Med* **324**: 1–7

Weidner N, Carroll PR, Flax J, Blumenfeld W and Folkman J (1993) Tumor angiogenesis correlates with metastasis in invasive prostate carcinoma. *Am J Pathol* **143**: 401–409

© Cancer Research Campaign 1999
